# Analysis of the High-Frequency Content in Human QRS Complexes by the Continuous Wavelet Transform: An Automatized Analysis for the Prediction of Sudden Cardiac Death

**DOI:** 10.3390/s18020560

**Published:** 2018-02-12

**Authors:** Daniel García Iglesias, Nieves Roqueñi Gutiérrez, Francisco Javier De Cos, David Calvo

**Affiliations:** 1 Arrhythmia Unit, Hospital Universitario Central de Asturias, 33011 Oviedo, Spain; danigarciaiglesias@gmail.com; 2 Instituto de Investigación Sanitaria del Principado de Asturias, 33011 Oviedo, Spain; 3 Grupo para la Modelización Matemática Avanzada (MOMA), Universidad de Oviedo, 33004 Oviedo, Spain; nievesr@uniovi.es (N.R.G.); fjcos@uniovi.es (F.J.D.C.)

**Keywords:** electrocardiographic analysis, wavelet transform, high-frequency content, sudden cardiac death

## Abstract

Background: Fragmentation and delayed potentials in the QRS signal of patients have been postulated as risk markers for Sudden Cardiac Death (SCD). The analysis of the high-frequency spectral content may be useful for quantification. Methods: Forty-two consecutive patients with prior history of SCD or malignant arrhythmias (patients) where compared with 120 healthy individuals (controls). The QRS complexes were extracted with a modified Pan-Tompkins algorithm and processed with the Continuous Wavelet Transform to analyze the high-frequency content (85–130 Hz). Results: Overall, the power of the high-frequency content was higher in patients compared with controls (170.9 vs. 47.3 10^3^nV^2^Hz^−1^; *p* = 0.007), with a prolonged time to reach the maximal power (68.9 vs. 64.8 ms; *p* = 0.002). An analysis of the signal intensity (instantaneous average of cumulative power), revealed a distinct function between patients and controls. The total intensity was higher in patients compared with controls (137.1 vs. 39 10^3^nV^2^Hz^−1^s^−1^; *p* = 0.001) and the time to reach the maximal intensity was also prolonged (88.7 vs. 82.1 ms; *p* < 0.001). Discussion: The high-frequency content of the QRS complexes was distinct between patients at risk of SCD and healthy controls. The wavelet transform is an efficient tool for spectral analysis of the QRS complexes that may contribute to stratification of risk.

## 1. Introduction

### 1.1. Physiological Basis

Cardiac malignant arrhythmias (MA) are the major cause of sudden cardiac death (SCD) in the general population, leading to great concerns in patients, physicians and health care systems everywhere [1]. Despite extensive research on arrhythmia mechanisms and treatment, which leads to highly effective therapies like implantable defibrillators, risk stratification continues to be a matter under debate. Thousands of patients are implanted a defibrillator every year worldwide, jet a minority of them will develop MA and benefits from the therapy. In addition, a significant part of them will develop adverse reactions like infections or lead fractures. In an attempt to maximize the benefit from the therapy and minimize adverse reactions, physicians would benefit from knowing a reliable source of data from the patients that allows for predicting future occurrence of MA, thus applying preventive measurements (i.e., implantable defibrillators) with the maximum of the sensitivity and specificity. Among a variety of technological tools for quantifying the risk, the stratification methods based on the surface electrocardiogram (ECG) have always been preferred by clinicians due to its widespread use, ease of access, simplicity in application and low associated cost. Under this perspective, the spectral analysis of the ECG emerges as potentially useful, with the opportunity to contribute to the risk stratification.

On the time domain analysis of the surface ECG, the fragmentation of the QRS complexes is defined as notches that disrupt the linear progression of the time-voltage series recorded from the body surface of the patients with an ECG sensor. Importantly, QRS fragmentation is gaining increasing attention as a risk marker for the development of SCD or Malignant Arrhythmias (MAs) that might help to stratification of risk in the clinical scenario [2,3]. In previous studies, the physiological basis of fragmentation has been related to delays in conduction of the electrical wavefronts through the myocardium, mostly secondary to the interposition of scar and fibrotic tissue [4,5]. The latter mechanism also explain the late potentials usually recorded from the myocardium of patients with ischemic cardiomyopathy [6,7] and other clinical entities with a high risk of SCD such as Hypertrophic Cardiomyopathy [8] or Dilated Cardiomyopathy [9]. The Brugada Syndrome (BrS) [10,11] is a particular case in which functional mechanisms promoting delayed conduction explain the occurrence of delay/late potentials in the absence of fibrotic tissue [12]. Both fragmentation of the QRS and delayed potentials have also been postulated as risk markers for SCD and MA in BrS patients [3,13].

Some studies have demonstrated a robust link between QRS fragmentation and late potentials [14]. Overall, fragmentation would represent a more prominent phenomenon that will allow for their identification by visual inspection of the time domain record of the QRS complex, while less prominent forms will be masked within the total energy/voltage of the normal myocardium, thus requiring signal processing for appropriate identification. The signal average is a classical method applied to time domain records [15,16]. Briefly, in this method a signal average is computed to increase the signal to noise ratio. Thereafter, a high pass filter allows the detection of low voltage/high-frequency potentials contained at the ending of the QRS complexes. However, the signal averaged ECG is highly dependent on noise, requires long time records, and displays low sensitivity for the detection of high-frequency content within the QRS (not at the end). In contrast, a variety of spectral methods may provide efficient analysis of the QRS signal, enabling identification of late potentials by their subrogates in the frequency domain: the high-frequency content [17]. As will be stated below, the Wavelet continuous transform might also have the advantage to efficiently locate the high-frequency content along the QRS complexes. The latter will be particularly interesting in characterizing dynamic process in which the electrical conduction delay in the myocardium varies according to physiological or interventional procedures [18].

### 1.2. The Wavelet Continuous Transform

As described by Roesch, the use of the Wavelet transform is a reasonable option to study periodic phenomena in time series, particularly in the presence of frequency changes over time [19]. The medical application of this technique for signal processing has been widespread since the early 1980s. The Morlet wavelet was first described in the early 1980s [20,21,22]. It is based on the Gabor transform [23], a Gaussian window sinusoid that allows decomposition of a signal in its frequency and phase content over time [19]. Unlike the Gabor transform, the Morlet wave maintains its shape through frequency changes, providing a separation of the contributions of different frequency bands without loss in temporal resolution [22]. In summary, the Morlet wavelet is defined by the following equation, where *w* represents the angular velocity or rotation, in radians per unit time:

(1) $$\psi \left(t\right)=\pi^{-1/4}e^{i\varpi t}e^{\frac{-t^{2}}{2}},$$

The Wavelet transform using the Morlet wavelet of a time series (*x_t_*) is defined as the convolution of the series with a set of daughter wavelets generated by the mother wavelet by time translation and scaled by *s*:

(2) $$Wave\left(\tau ,s\right)=\sum_{t}x_{t}\frac{1}{\sqrt{s}}\psi \times \left(\frac{t-\tau }{s}\right),$$

The position of the daughter wavelet in the time domain is determined by the displacement of the time parameter in increments of *dt*. The local amplitude of any periodic component of the time series and how it evolves over time can be obtained from the module of its wavelet transform [19]. The square of the amplitude constitutes the density of the wavelet energy in the time-frequency domain and is called the energy spectrum of the wave [24]:

(3) $$Power\left(\tau ,s\right)=\frac{1}{s}\times \left|Wave{\left(\tau ,s\right)}^{2}\right|,$$

### 1.3. The Use of the Wavelet Transform for the Study of the Surface Electrocardiogram

The Wavelet transform applied to the surface ECG has been analyzed in variety of clinical scenarios. In the works by Gramatikov et al., the continuous Wavelet transform was used to detect high-frequency content along the QRS in patients with acute ischemia, either provoked during a coronary angioplasty procedure (mechanical occlusion of the artery lumen), or spontaneously occurring in patients with acute ischemic heart disease (angina pectoris) [25,26]. An increase in the high-frequency content during angioplasty (frequency range: 16–200 Hz) and during the acute ischemic episodes (frequency range: 20–100 Hz) was observed in these studies, and subsequently normalized once the ischemia disappeared. Similarly, Magrans et al., studied patients during a coronary angioplasty procedure [27]. They found that, at the time of the angioplasty, the high-frequency content increased. Remarkably, those changes were more pronounced at the terminal part of the QRS signal, thus suggesting displacements of the high-frequency content along the QRS that must be consider in terms of the signal processing and the interpretation of data. Another interesting field for study, in which the wavelet transform of the QRS complexes has been successfully used, is to analyze patients with specific electrical conduction delays requiring resynchronization therapy. With this regard, it has been postulated that the use of the Wavelet transform might help to discern patients that will be unresponsive to the therapy, thus avoiding costs and potential adverse events [28,29].

However, the number of patients included for analysis in those studies is low and the possibility of implementing automatic systems for the analysis of QRS complexes has not been entirely evaluated. The utility of the Wavelet transform to analyze the high-frequency content and to predict the risk of MA or SCD have been poorly studied. Only the work of Murata et al., has shown an increase in the high-frequency content of patients with MA or SCD compared to those without [17,30,31].

### 1.4. Pan-Tompkins Algorithm for Automatic Detection of QRS Complexes

The detection of QRS complexes is the first step for any automatic ECG analysis. There are many methods for this purpose, with generally good performance, although each method has situations where it fails. The algorithm published in 1985 by J. Pan and W. Tompkins is one of the most used and allows the correct detection of the dominant peaks (R wave) of the QRS complexes [32]. In later works the effectiveness of this algorithm has been evaluated and compared with other algorithms [33], just confirming an adequate performance [34,35]. Briefly, the Pan-Tompkins algorithm is based on 4 sequential steps: (i) differentiation, (ii) squared elevation, (iii) integration with a moving window and finally, (iv) selection of the detection threshold to select the R waves of the QRS complexes.

### 1.5. Hypothesis and Objectives

Hypothesis: The high frequency content of the surface ECG display distinctive characteristics in patients under a risk of MA and SCD.

Objective 1: To evaluate the ability of the Wavelet transform for the analysis, quantification and temporal localization of high frequency content in QRS complexes, both in healthy controls and in patients with a history of SCD or MA.

Objective 2: To analyze the contributing power and time distribution of the high frequency content within the QRS complexes in patients and healthy controls in order to study differential patterns between them.

## 2. Materials and Methods 

### 2.1. Study Population

The group of patients was obtained from a consecutive registry of 42 patients with prior history of Sudden Cardiac Arrest (SCA) or MA (patients), who came to our cardiac electrophysiology laboratory for diagnostic or therapeutic purposes. The term SCA was used to describe sudden death cases in which specific resuscitation records were available or the individual had survived the cardiac arrest event [35]. SCD was defined as an unexpected death without obvious extracardiac cause, occurring with a rapid witnessed collapse, or if un-witnessed, occurring within 1 h after the onset of symptoms. SCD was considered probable if occurred unexpectedly without obvious extracardiac cause within the previous 24 h. In any situation, the death should not occur in the setting of a prior terminal condition, such as a malignancy that was not in remission or end-stage chronic obstructive lung disease [35]. MA were defined as sustained life-threatening ventricular arrhythmia (Ventricular Tachycardia or Ventricular Fibrillation), which finished spontaneously or with treatment (pharmacological or electrical). A control group of 120 healthy individuals (controls) were analyzed. In all of them cardiac examination was performed to rule out any cardiac disease.

For the collection of electrocardiographic data the ethical committee approval was first obtained (Asturias regional ethical committee for clinical research, project number 35/2013). In addition, the investigations were carried out following the rules of the Declaration of Helsinki of 1975, revised in 2008.

### 2.2. Collection of Electrocardiographic Data: The ECG Sensor

**Figure 1:**
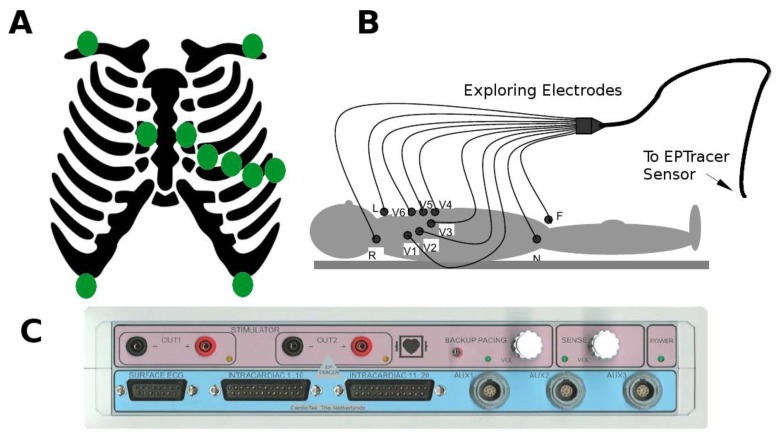
Electrocardiogram (ECG) recording and characteristics of the EPTracer sensor. (**A**) Schematic diagram representing the standard position of the exploring electrodes (green dots) on the body surface. (**B**) According to the position of the exploring electrodes on the body surface, and the configuration of pairs for computing differences in potential, the ECG signals receive standardized names. L, R, F, V1, V2, V3, V4, V5, and V6 constitute the differences in potential between the corresponding electrode and an indifferent electrode (central terminal of Wilson). In addition, three ECG signals are computed as the algebraic sum of potentials at different pairs of electrodes (lead I = L + R; lead II = R + F; Lead III = L + F). (**C**) EPTracer sensor front panel.

The standard ECG (12 leads) of the patients included in the study were digitally collected with the commercially available ECG sensor EPTracer^®^ from CardioTek© Electrophysiological Measurement System, Maastricht (here in after the EPTracer sensor). The EPTracer sensor is a computerized system designed for both clinical and experimental electrophysiological studies, allowing real-time visualization of the ECG signal and continuous storage (12 bit resolution, up to 1 kHz sampling rate, and a maximal differential input-voltage of +/−5 mV recording). As displayed in Figure 1, panels A & B, the potential at different sites of the body surface was explored by 10 exploring electrodes distributed according to clinical standards. The opposite extremes of the electrodes were connected to a multi-channel amplifier (panel C), where potential differences (ECG signals or leads) were computed between pairs of electrodes or calculated against an indifferent electrode (central terminal of Wilson) [36,37]. ECG signals underwent filtering at the EPTracer sensor (linear-phase digital band-pass filter: 0.05–150 Hz to suppress low-frequency noise that results from baseline wander, movement, and respiration, and higher-frequency noise that results from muscle artifact, and power-line or radiated electromagnetic interference) [37] and amplification. A notch filter was also applied at the 50 Hz frequency, in order to eliminate the power-line interference.

### 2.3. Extraction of QRS Complexes Using a Modified Pan-Tompkins Algorithm

**Figure 2:**
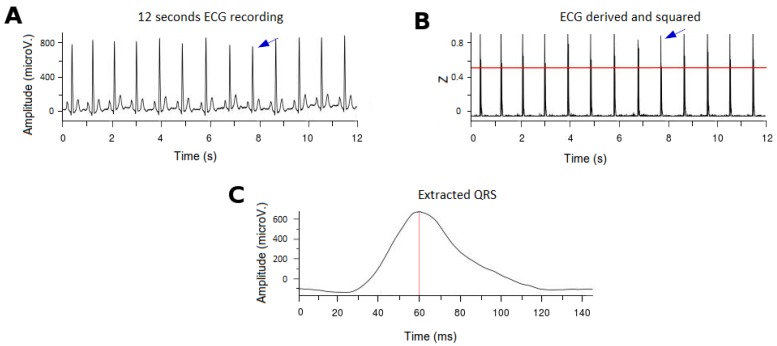
Example of the ECG processing and the QRS extraction. (**A**): Surface ECG (12 consecutive seconds). The blue arrow indicates a QRS complex. (**B**) Signal after derivation, square elevation and typification. The 0.6 threshold to detect the QRS complexes is denoted (red line). The blue arrow indicates a QRS complex after processing. (**C**) Example of an extracted QRS complex. The *tMaxQRS* is indicated by the red line. ECG: electrocardiogram.

For the purpose of the study, the ECG records were performed continuously for at least 12 consecutive seconds (Figure 2A). To extract the QRS complexes, we propose a modified algorithm of the originally described by Pan-Tompkins [32]. As previously described, the latter consisted of four consecutive steps: differentiation, squared elevation, detection threshold calculation, and correction by local maxima. However, in order to make spectral comparisons between subjects that affected the amplitude and the timing of the high frequency content, there is a need for: (i) normalizing the amplitude of the QRS signal; and (ii) select a stable reference for timing. After normalization, we would need also to select appropriate new thresholds for QRS detection. The latter is the rationale for the modifications that we introduced in the originally described Pan-Tompkins algorithm, summarized as follows (see also Figure 2):

First, we normalized the ECG signal to allow appropriate comparisons between leads and patients.

In order to determine the threshold for detection of the R-wave in the QRS complexes (after differentiation and squared elevation of the signal), the 99.5 percentile of the signal (*P99.5*) was calculated. All the extreme values (defined as those greater than *P99.5*) were removed and the signal was typified over the value of *P99.5* (Figure 2B). A threshold of 0.6 defined the time points where there were QRS complexes (*tQRS*; red line in Figure 2B).

A temporal correction was performed for the morphology of the QRS complexes. To do this, the point of the QRS complex with a higher positive voltage value in the V6 derivation was selected (*tMaxQRS*; red line in Figure 2C), because clinically it is the lead in which the peak of the R wave is better defined.

Finally, to subtract the QRS complexes for analysis a window of 145 ms around the *tMaxQRS* point was selected (from 60 ms before *tMaxQRS* to 85 ms after *tMaxQRS*; Figure 2C).

In order to evaluate the algorithm, we compared our modified algorithm’s performance against other published algorithms. For this purpose we used the MIT-BIH Arrhythmias Database [38], and calculated the Sensitivity (Se), Positive Predictive Value (PPV), Error (Er), Total Beats (TB), False Positives (FP) and False Negatives (FN), as described in previous methods [32,39,40,41,42].

### 2.4. Wavelet Continuous Transformation for the Analysis of the High-Frequency Content

**Figure 3:**
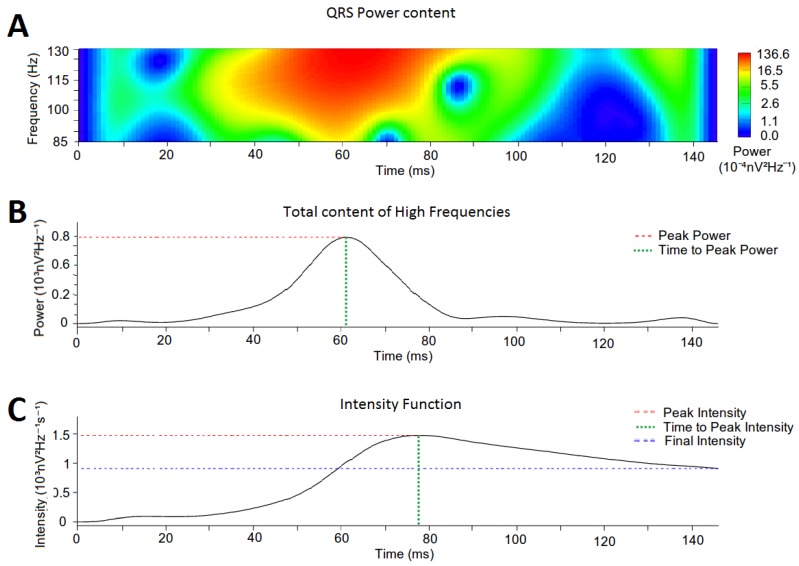
Example of a Wavelet continuous transform on a QRS complex (frequency range: 85–130 Hz). (**A**) Power spectrum of the QRS complex. (**B**) Cumulative power of the high-frequency content at each time epoch. The red dotted line marks the *Peak Power* and the green dotted line marks the *Time to Peak Power*. (**C**) Intensity Function. *Final Intensity* is marked by blue dotted line; *Time to Peak Intensity* is marked by the green dotted line; and *Peak Intensity* is marked by the red dotted line.

The time-frequency data of each QRS complex were collected using the Wavelet transform (Morlet wavelet). According to previous definitions of high frequency content in other works [14,15,16,17], data were analyzed in the defined range of high frequencies (85–130 Hz), with an upper period of 11.5 ms and lower period of 7.7 ms (Figure 3A). A temporal definition of 1 kHz and a frequency resolution of 1/125 suboctaves were used. Calculations for the Wavelet Continuous Transform were performed with the WaveletComp library [19] for R [43].

### 2.5. Quantification of High-Frequency Content in the QRS Complexes

To analyze the distribution of the high-frequency content along the QRS signal, we computed the cumulative power contained at each time epoch of the QRS complex (Figure 3B). From the obtained distribution we defined (i) the *Peak Power* as the highest cumulative power of the high-frequency content (red dotted line in Figure 3B); (ii) the *Time to Peak Power* as the time epoch where Peak Power was reached (green dotted line in Figure 3B); and (iii) the *Total Power* as the area under the curve of the whole power function.

In addition, we calculated the relative contribution of the high-frequency content to the total power of the QRS complexes as a function of time: (i) at the initial part of the QRS complex (prior to *tMaxQRS*) named the *Initial High Frequency Contribution*; and (ii) at the final part of the QRS (after *tMaxQRS*) named the *Final High Frequency Contribution*. The ratio between the initial and final contribution was defined as the *High Frequency Contribution Ratio*.

### 2.6. Intensity Analysis

For a better description of the distribution of the high-frequency content along the QRS complex, the spectral intensity of the high-frequency content throughout the entire QRS complex is calculated. Intensity was defined as the instantaneous average of cumulative power and computed according to the following equation:

(4) $$Intensity\left(t\right)=\frac{1}{dt}\times \sum_{i=1}^{t}\left(Power_{i}\right),$$

where *Power_i_* is the average high-frequency power in *t = i* and *dt* is the time elapsed until each time epoch. On the spectral intensity function we defined: (i) the *Peak Intensity* as the highest intensity in the intensity function (red dotted line in Figure 3C); (ii) the *Time to Peak Intensity* as the time epoch where *Peak Intensity* was reached (green dotted line in Figure 3C), (iii) the *Final Intensity* as the intensity value at the ending point of the intensity function (blue dotted line in Figure 3C); and (iv) the *Total Intensity* as the area under the curve of the whole function.

### 2.7. Statistical Analysis

Initially, a descriptive statistical analysis of the baseline variables of the patients was performed to characterize the study population. Categorical variables are expressed as percentage and the continuous quantitative as Mean ± Standard Error. For the contrast between patients and controls, a Chi square test was performed for the categorical variables and the Student’s T test for the continuous variables, in all cases with a significance level of 0.05. The quantification of the parameters used to analyze the high-frequency content along the entire QRS complex, as well as the spectrogram intensity in the high-frequency range is expressed as Mean ± Standard Deviation. For the contrast between the patients and the controls we used a Student’s T test. Statistical calculations were performed with the statistical software R [43].

## 3. Results

### 3.1. Population Characteristics

We analyzed data from 162 individuals (120 healthy controls and 42 patients). Table 1 summarizes clinical characteristics. No statistical differences between both groups were found. Ischemic Heart Disease (28.57%) and BrS (26.19%) were the most frequently observed pathology in patients. All of them had suffered at least an episode of MA, and 11.9% had experienced an episode of SCA.

### 3.2. Comparative Analysis between Different QRS Detection Algorithms

As previously stated, the proposed modifications in the Pan-Tompkins algorithm were designed to allow appropriate comparisons of the high frequency content between different subjects. Although improving detection features was out of our goal, we consider appropriate to display the performance of the modified algorithm in comparison with others. Table 2 display that Sensitivity and Positive Predictive Values of our modified algorithm remain in the range of those previously published for other methods. However, the Sensitivity and the Positive Predictive Value are slightly lower for our modified Pan-Tompkins in comparison with the originally described. 

### 3.3. Analysis of the High-Frequency Content

**Figure 4:**
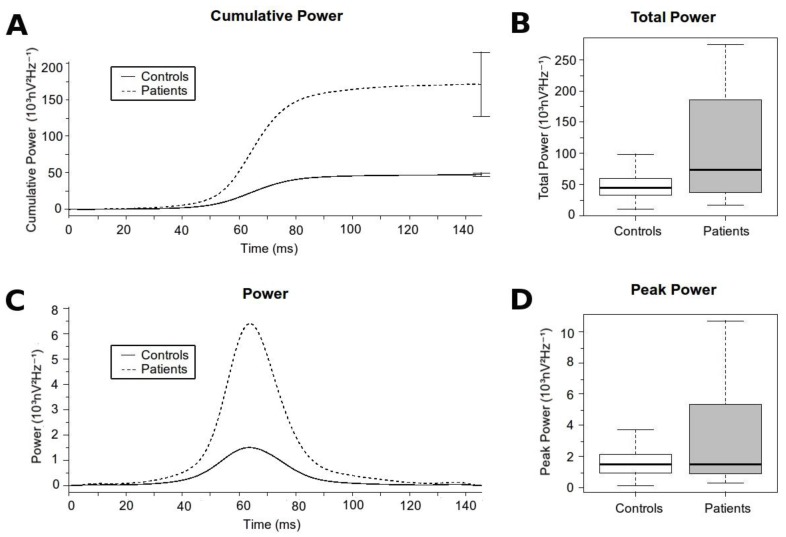
High-frequency content along the QRS Complex. (**A**) Cumulative high-frequency content for each group at each time epoch. The final error bars represents the Total Power with its standard error for each group. (**B**) Boxplot comparing the Total Power. (**C**) Time distribution of the high-frequency content for each group at each time epoch. (**D**) Boxplot comparing the *Peak Power.*

**Figure 5:**
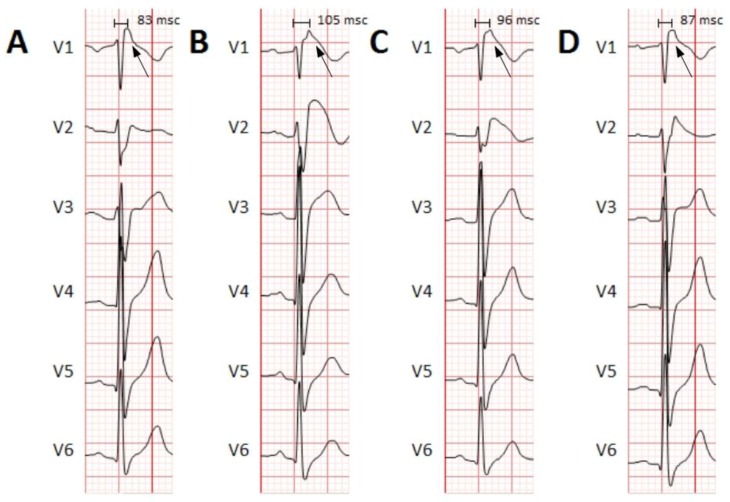
Example of different QRS complexes at different times in a patient with BrS. The precordial leads (V1 through V6) are shown. (**A**) Basal ECG, before Flecainide administration. Flecainide is an antiarrhythmic drug with major effects on cardiac cell’s depolarization. Due to its effects on the cardiac sodium channel (main alteration in BrS), it is used in patients with suspected BrS to unmask the characteristic electrocardiographic pattern. No Brugada pattern is observed. (**B**,**C**) ECG showing a Brugada pattern immediately and a few minutes after Flecainide administration. A moving wave becomes apparent with increasing delay (black arrows and time intervals). (**D**) Resumption to basal conditions demonstrating the returning of the moving wave to more proximal parts of the QRS. BrS: Brugada syndrome. ECG: electrocardiogram.

Overall, patients had significantly higher high-frequency content along the QRS complex than controls (data summarized in Table 3 & Figure 4). The *Total Power* and the *Peak Power* were significantly higher in patients compared with controls. The *Time to Peak Power* was also prolonged in patients with regard to controls, thus demonstrating differences not only in the absolute values, but also in the distribution of the high-frequency content along the QRS. However, when analyzing the relative contribution of the high-frequency content there were no differences in the *High Frequency Contribution Ratio* between patients and controls. That probably means a global displacement of the total frequency content to more distal positions in patients compared with controls. The latter is well exemplified in a patient with BrS in which, at different states of the pathology, the time domain record of the QRS complex displayed progressively delayed components (moving waves; Figure 5, black arrow) at the terminal part of the QRS. This case illustrates that as the ECG record becomes more pathological, the position of the moving wave is located at more delayed positions of the QRS. With regression to normal ECG tracings, the moving wave returns to more proximal positions.

### 3.4. Intensity Analysis

**Figure 6:**
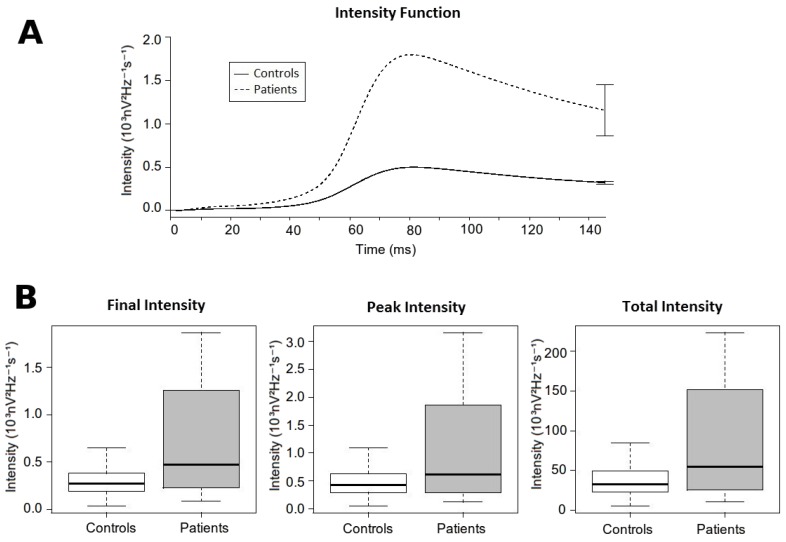
Intensity analysis. (**A**) Intensity function. It shows the value of the intensity function for each group in each time epoch. The final error bars represent the Final Intensity with its standard error for each group. (**B**) Boxplot comparing different measures of the intensity function between both groups. From left to right: *Final intensity*, *Peak intensity* and *Total Intensity.*

Overall, patients displayed higher intensity of the high-frequency content along the QRS complex than controls (data summarized in Table 4 & Figure 6). The *Final Intensity*, the *Peak Intensity* and the *Total Intensity* were significantly higher in patients compared with controls. In addition, the *Time to Peak Intensity* was reached later in patients when compared with controls.

## 4. Discussion

The results of our study show that the wavelet transform of the QRS complexes allows for appropriate characterization of the high-frequency content that exert a differential behavior between healthy individuals and patients affected by severe cardiac arrhythmias leading to SCD. In our cohort of patients, we demonstrate that the relative contribution of the high frequencies to the spectral content is higher than in healthy controls, and behave with a slightly delay in their appearance at the QRS complexes. Overall, the absolute values and the distribution of the of high-frequency content may be of prognosis significance in patients with different pathologies, which in conjunction with the analysis of other clinical variables, like cardiac syncope and structural abnormalities, might contribute to better stratification of risk and more appropriate measurements to take in order to reduce the risk of SCD. In addition, the analysis proposed in our work is feasible for an automatized, on-line analysis of ECG records that would allow quick evaluation of patients.

The Wavelet continuous transform has been proposed as a useful technique for studying the frequency power spectrum, and especially to study the temporal distribution of the high-frequency content along QRS complexes. In this sense, it is important to remark how this constitutive part along the QRS may present a dynamic behavior with time and also change under different clinical situations even within the same patient. At different states of the pathology the morphology of the ECG may change, which might make diagnosis of those patients difficult [18]. In addition, those changes on the ECG morphology may also change the distribution of the frequency content along the QRS, depending on the severity of the disease or the administration of various drugs. For example, in Figure 5 we display the case of a patient diagnosed with BrS. The ECG records show how there is a displacement of late potentials towards a later position within the QRS complex as the ECG demonstrates more pathological conditions (from Brugada type III to Brugada Type I). This dynamic behavior makes it important to consider analytical techniques that are able to track the displacements in the frequency content in order to increase precision and sensitivity. We also previously demonstrate that ventricular fibrillation, the most lethal arrhythmia causing SCD in patients, is characterized by a highly dynamical behavior in the frequency content, but with maintenance of a hierarchical organization at the phase spectrum [13]. The latter allows for spatial location of the sources that eventually maintain the arrhythmia, and pave the way for automatized analytical methods able to efficiently track those dynamical changes and displacements of the frequency content that would help for developing more efficient clinical procedures.

Compared with other techniques focused on the time or frequency domain, we consider that the wavelet transform may provide significant advantages. Although firstly reported, the analysis of QRS fragmentation on the time domain may be less useful than the analysis of the high-frequency content to detect patients at risk for SCD [2,8,9]. The main limitation of the analysis of fragmentation is that it is based on a subjective classification, with no absolute measurements, and thus it is more difficult to standardize. Also they are less sensitive than frequency domain analysis [44]. In addition, unlike other techniques for the analysis of the frequency power spectrum (i.e., Fast Fourier Transform), the time-frequency analysis (i.e., Wavelet transform) also allows for the location of high-frequency content along time [45], which can help to identify significant displacements as discussed before. There are other techniques for time-frequency analysis, mainly the signal-averaged electrocardiogram [45], which is one of the first described techniques for high-frequency analysis in QRS complexes [15,16]. As stated at the introduction section, this technique may fail in situations with high noise to signal ratio, it requires long ECG records, and it is only useful in the terminal part of the QRS, not in the whole QRS complex [46]. Spectral methods provide a more efficient analysis of the QRS signal [17] and we have found the Wavelet transform particularly useful in our patients.

## 5. Conclusions

The high-frequency content of the QRS complexes distinctively behaves between patients at high risk of SCD, and healthy controls. The wavelet transform is an efficient tool for spectral analysis of the QRS complexes that allows for quantifying the differential behavior between both studied groups. Future research is needed to clarify how the proposed technique may contribute to stratification of patient risk. 

## 6. Limitations

Differences between patients with and without MA/SCA were not evaluated, thus we cannot ascertain the predictive capabilities in patients in primary prevention. Patient groups had a higher percentage of men than the control group. Because there may be some differences in QRS characteristics associated with gender, it could explain, to some extent, the differences observed between groups.

Overall, to analyze the global utility of the Wavelet transform, comparisons with other techniques are required. In order to develop an automatic method for quantification and characterization of high-frequency content there is need for combining the relevant metrics (high frequency content timing and magnitude) using a machine learning technique, and assessing its performance. Both will be desirable but were not tested/performed in our work.

## Figures and Tables

**Table 1:** Population characteristics.

Variables	Controls (*n* = 120)	Patients (*n* = 42)
Age (years)	53.3 ± 14.37	59.6 ± 18.26
Male (N/%)	67 (55.8)	34 (80.95)
Cardiomyopathy (N/%)	0 (0)	42 (100)
Ischemic	NA	12 (28.57)
Idiopathic	NA	4 (9.52)
BrS *	NA	11 (26.19)
Others	NA	15 (35.71)
Arrhythmic Events (N/%)	0 (0)	42 (100)
SCA *	NA	5 (11.9)
MA *	NA	42 (100)

* BrS: Brugada Syndrome; SCA: Sudden Cardiac Death; MA: Malignant Arrhythmias.

**Table 2:** Comparative analysis between different QRS detection algorithms.

Algorithm	Database	Se (%)	PPV (%)	Er	TB	FP	FN
J. Pan et al. (1985) [32]	MITDB	99.75	99.53	0.675	116137	507	277
J.P. Martinez et al. (2004) [42]	MITDB	99.8	99.86	0.34	107567	153	220
Z. Zidelmal et al. (2012) [39]	MITDB	99.64	99.82	0.54	109494	193	393
R. Tafresi et al. (2014) [40]	PTBDB	99.06	98.9	N/A	N/A	N/A	N/A
M. Yochum et al. (2016) [41]	CinCC11	99.87	91.71	N/A	N/A	N/A	N/A
Present Work	MITDB	98.45	96.67	3.53	114654	2567	1125

* Se: Sensitivity; PPV: Positive Predictive Value; Er: Error; TB: Total Beats; FP: False Positives; FN: False Negatives.

**Table 3:** Summary of high-frequency analysis.

Variables	Controls (*n* = 120)	Patients (*n* = 42)	*p*
Peak Power (10^3^nV^2^Hz^−1^)	1.709 (± 1.13)	7.033 (± 15.09)	0.028
Time to Peak Power (ms)	64.768 (± 5.868)	68.952 (± 7.609)	0.002
Total Power (10^3^nV^2^Hz^−1^)	47.298 (± 26.129)	170.782 (± 282.714)	0.007
Initial High Frequency Contribution (10^3^nV^2^Hz^−1^)	2.012 (± 1.21)	5.409 (± 4.97)	<0.001
Final High Frequency Contribution (10^3^nV^2^Hz^−1^)	45.287 (± 25.601)	165.463 (± 280.608)	0.008
High Frequency Contribution Ratio	0.053 (± 0.034)	0.069 (± 0.049)	0.059

**Table 4:** Summary of the intensity analysis.

Variables	Controls (*n* = 120)	Patients (*n* = 42)	*p*
Peak Intensity (10^3^nV^2^Hz^−1^s^−1^)	0.506 (± 0.296)	1.854 (± 3.389)	0.014
Time to Peak Intensity (ms)	82.116 (± 5.103)	88.738 (± 9.461)	<0.001
Total Intensity (10^3^nV^2^Hz^−1^s^−1^)	39.024 (± 21.574)	137.128 (± 233.521)	0.001
Final Intensity (10^3^nV^2^Hz^−1^s^−1^)	0.32 (± 0.177)	1.155 (± 1.91)	0.007
